# The Importance of Primary Care Cognitive Evaluation in Detecting an Atypical Presentation of Normal Pressure Hydrocephalus

**DOI:** 10.7759/cureus.62166

**Published:** 2024-06-11

**Authors:** Miriam Rivkin, Sumona Banerjee, Torrey Guan, Christian Hendrix

**Affiliations:** 1 Internal Medicine, Saint Louis University School of Medicine, St. Louis, USA

**Keywords:** normal pressure hydrocephalus, magnetic resonance imaging (mri), auditory hallucinations, primary care medicine, cognitive assessment

## Abstract

Normal pressure hydrocephalus (NPH) is a syndrome that characteristically presents with progressive gait impairment, cognitive deficits, and urinary urgency or incontinence. We present a case of a 54-year-old male with a past medical history of alcohol use and no primary care provider with new-onset auditory hallucinations. The patient was found to have a marked enlargement of the supratentorial and infratentorial ventricles on both computed tomography (CT) and magnetic resonance imaging (MRI) and an opening pressure of 21 on the lumbar puncture, concerning for NPH. Clinically, there were signs of cognitive impairment due to memory and cognitive function loss, but the patient lacked gait disturbances or incontinence. Although not common, NPH may present with auditory hallucinations or delusions, as seen with our patient. In this case report, we emphasize the importance of annual cognitive assessments in order to evaluate atypical psychiatric manifestations of neurological disorders. Because clinical symptoms are more likely to be reversible when recognized early in the clinical course and the progression of these symptoms can be prevented with the placement of a ventriculoperitoneal (VP) shunt, it is of utmost importance to accurately recognize and diagnose NPH as early as possible. We also discuss the less commonly known markers of NPH on MRI.

## Introduction

Normal pressure hydrocephalus (NPH) is a syndrome that characteristically presents with progressive gait impairment, cognitive deficits, and urinary urgency or incontinence. Idiopathic normal pressure hydrocephalus is the most common form in adults, and other secondary causes may include infection, hemorrhage, traumatic brain injury, or radiation [1]. In the United States, the prevalence of idiopathic NPH is estimated to be 700,000 persons. Of these, 33,808 persons are 70-79 years old, and 669,178 persons are 80 years or older [2]. Although the exact mechanism of idiopathic NPH is unclear, several mechanisms have been described such as the hyperdynamic flow of cerebrospinal fluid (CSF) in the aqueducts, increased CSF pulse pressure, or even abnormal CSF reabsorption mechanisms. This increased intracranial pressure leads to its constellation of symptoms via compression on key structures within the nervous system such as the fibers of the corticospinal tract in the corona radiata that supply the legs, the inner table of the calvarium leading to dementia, and the periventricular sacral fibers leading to the loss of voluntary bladder control [1]. Symptoms of NPH may worsen over time if the condition is left untreated, potentially leading to seizures and severe dementia [1].

## Case presentation

A 54-year-old male with a past medical history of alcohol use and no primary care provider presented to the police station with new-onset auditory hallucinations. Upon arrival at the emergency department, vitals were normal. Laboratory evaluation for metabolic, infectious, and toxic causes of encephalopathy was negative. Despite a history of drinking about 2-6 beers daily, he was not clinically intoxicated. His blood alcohol content returned at <0.010 g/dL, and his urine toxicology screening was negative. Computed tomography (CT) of the patient's head showed marked enlargement of the supratentorial and infratentorial ventricles, concerning for NPH.

**Figure 1:**
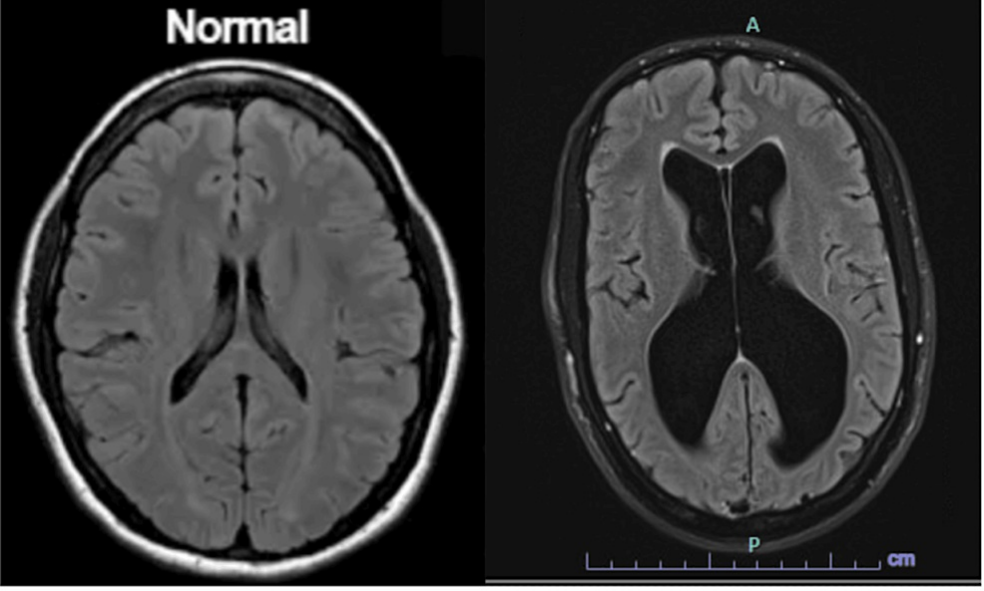
Normal ventricular size versus the MRI of our patient's ventricles exhibiting severe ventricular dilation causing normal pressure hydrocephalus MRI: magnetic resonance imaging

During his admission, he remained alert and oriented to person, place, and time. Magnetic resonance imaging (MRI) of the brain re-demonstrated findings concerning for NPH with no evidence of acute intracranial findings or abnormal enhancement (Figure 1). The patient denied a history of gait disturbances or incontinence. Clinically, there were signs of cognitive impairment due to memory and cognitive function impairment. Results from lumbar puncture were significant for opening pressure at 21 cm H_2_O but did not show signs of xanthochromia or abnormal fluid content. Based on these results and the patient's presentation, it was determined that he would benefit from shunt placement by neurosurgery.

Two weeks after discharge, this patient was seen in the neurosurgery clinic for shunt placement. He denied any new neurological symptoms; however, at this time, he did endorse a history of imbalance and occasional incontinence.

The patient's auditory hallucinations were remarkable for a voice telling him people were after him, and therefore, he did not feel safe at home. He was evaluated for psychiatric conditions; however, disorders such as schizophrenia and alcoholic hallucinosis were subsequently ruled out given that the patient was not experiencing any other symptoms required for diagnosis.

## Discussion

Although normal pressure hydrocephalus is classically described by the triad including dementia, gait disturbance, and urinary incontinence, atypical psychiatric symptoms may present as the predominant clinical finding. The evaluation of NPH requires ruling out metabolic, infectious, and intracranial lesions and other cerebrovascular diseases, which was done for this patient. Because clinical symptoms are more likely to be reversible when recognized early in the clinical course and the progression of these symptoms can be prevented with the placement of a ventriculoperitoneal (VP) shunt, it is of utmost importance to accurately recognize and diagnose NPH as early as possible.

Multiple studies have previously explored the association between NPH and various psychiatric presentations, possibly as a result of alterations to the central neurotransmitter activities [3]. NPH may present with auditory hallucinations and delusions, as seen in this particular case, or with psychosis, personality change, and mania [4]. However, a commonly overlooked aspect of these studies is the exploration of how to best prevent the progression of NPH in these patients with psychiatric or behavioral symptoms. One key component to emphasize is the importance of establishing care with a primary care physician (PCP). By doing so, patients will be able to be screened for cognitive impairment during annual visits. For example, for patients >65 years old, annual wellness visits under Medicare cover cognitive assessments. In our case, the patient did have insurance but did not establish care with a PCP and thus was not appropriately evaluated for cognitive dysfunction despite his sister stating that she began to notice changes in his cognitive status for the past two years. This delay in recognition and treatment may impact a full recovery following a lumbar puncture. Another important aspect to emphasize is for PCPs to be conscious of psychiatric findings as indicative of NPH. As such, a careful workup to screen for indications of brain injury is necessary.

The hallmark of NPH includes ventriculomegaly without sulcal enlargement and without obstruction or evidence of third or fourth ventricle enlargement. Additional imaging findings include an Evans index of >0.3 ratio of the frontal horn of the lateral ventricle over the largest inner diameter of the skull on the same axial level. NPH can also be categorized by the callosal angle. MRI is the preferred imaging as it delineates specific markers of NPH that otherwise cannot be found on CT scans. One marker that can be identified on MRI is disproportionately enlarged subarachnoid space hydrocephalus (DESH). DESH is characterized by ventriculomegaly, tight high-convexity and medial subarachnoid space, the disproportionate enlargement of the Sylvian fissures, and focally dilated or entrapped sulci without adjacent cortical atrophy [5]. In addition to these markers, MRI is also able to detect periventricular white matter changes, which may be associated with the degree of cognitive impairment in NPH patients. MRI can also be used to differentiate NPH-caused psychiatric conditions from other causes, such as Alzheimer's disease (AD). In two series, medial temporal atrophy, as manifested by either more dilated perihippocampal fissures or a lower hippocampal volume, was significantly greater in patients with AD compared with NPH [6].

## Conclusions

The atypical presentation of normal pressure hydrocephalus in our case demonstrates the importance of cognitive screening in elderly populations. For those with concern for NPH, this case demonstrates the need to order appropriate imaging to identify the correct markers of NPH. As symptoms of NPH are reversible, it is of utmost importance both for patients to be cognizant of the benefits of establishing care with their PCP and for providers to be aware of the unique psychiatric clinical presentations that may manifest with normal pressure hydrocephalus.
